# Asymmetric arene hydrogenation: towards sustainability and application

**DOI:** 10.1039/d3cs00329a

**Published:** 2023-07-10

**Authors:** Lukas Lückemeier, Marco Pierau, Frank Glorius

**Affiliations:** a Universität Münster, Organisch-Chemisches Institut, Corrensstraße 36 48149 Münster Germany glorius@uni-muenster.de

## Abstract

(Hetero)aromatic compounds are vastly available and easy to functionalise building blocks in the chemical industry. Asymmetric arene hydrogenation enables direct access to complex three-dimensional scaffolds with (multiple) defined stereocentres in a single catalytic process and, by this, the rapid installation of molecular complexity. The potential usage of hydrogen from renewable sources and perfect atom economy bears the potential for sustainable and broadly applicable transformations to valuable products. The aim of this review is to present the state-of-the-art in transition-metal catalysed asymmetric hydrogenation of (hetero)arenes, to highlight recent advances and important trends and to provide a broad overview for the reader.

## Abstract 2

This review summarises the state-of-the-art in transition-metal catalysed asymmetric hydrogenation of (hetero)arenes and highlights recent advances with a special focus on sustainability while also addressing its shortcomings.

## Graphical abstract

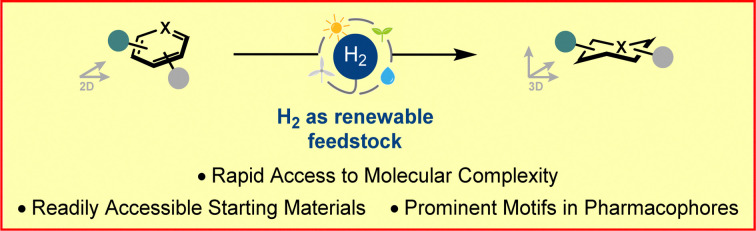

## 1. Introduction

Arenes and heteroarenes belong to the most ubiquitous structural motifs in natural products, agrochemicals and pharmaceuticals.^1–5^ They are also versatile building blocks since they are widely accessible starting materials, can bear multiple heteroatoms and can be modified easily by numerous synthetic methodologies (*i.e.* cross coupling, S_N_Ar, aromatic halogenation), especially for drug synthesis. This is also reflected by the fact that the fractional sp^3^ character (Fsp3) of molecules published in the Journal of Medicinal Chemistry between 1959 and 2009 decreased by ∼10%.^6^

The saturated aromatic analogues are less prominently represented, however they are at least as important for *i.e.* drug discovery. Beneficial correlations have been shown between important structural elements, such as high Fsp3, increased molecular complexity or aliphatic ring count, and the clinical success of drug candidates.^7–9^ In contrast to that, a high aromatic ring count can be associated with certain parameters like poor solubility in aqueous media, high lipophilicity or decreased protein binding.^10,11^ For this reason, saturated (hetero)cyclic scaffolds are featured in a vast number of top-selling small molecule drugs, frequently with precise stereochemistry.^12^ However, the synthesis of aliphatic rings can be challenging and step-intensive. Consequently, it has been of high interest in recent years to develop efficient pathways to access these scaffolds in an efficient manner.^13–15^

**Fig. 1:**
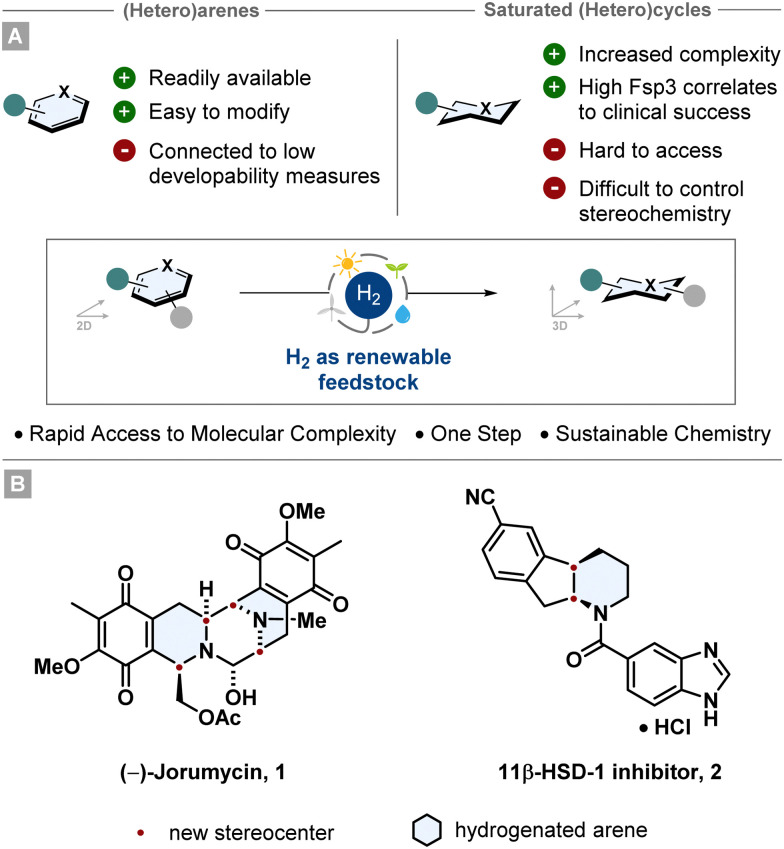
Hydrogenation as an environmentally benign method to provide direct access to diverse saturated carbo- and heterocyclic motifs.

Arguably the most sustainable and powerful way to synthesise stereochemically well-defined aliphatic (hetero)cycles is the asymmetric hydrogenation of (hetero)arenes. Starting from broadly accessible and easily modifiable (hetero)arenes, multiple stereocentres can be set in a single synthetic operation giving rise to complex and enantioenriched (hetero)cyclic scaffolds (Fig. 1). Since this catalytic transformation only adds molecular hydrogen to a molecule, the atom economy is perfect rendering hydrogenation as an intrinsically green reaction.^16^ Further features such as the use of renewable feedstocks and the reduction in waste and step-count emphasise the environmentally benign character. Taking advantage of this strategic value, asymmetric (hetero)arene hydrogenation is employed in numerous industrial processes and total syntheses. Beautiful recent examples are the total syntheses of (−)-jorunnamycin A and (−)-jorumycin 1 by the Stoltz group^17^ and the multikilogram synthesis of a promising diabetes drug candidate 2 by chemists of Boehringer Ingelheim (Fig. 1).^18^ Both routes utilise an asymmetric heteroarene hydrogenation as a key step to construct the cores of the complex target structures. This tutorial aims to convey the reader important methods and principles of asymmetric transition metal catalysed arene hydrogenation, highlight historically significant and recently developed catalyst systems and give an outlook into the future of the field. Notably, asymmetric organocatalytic hydrogenation of arenes is also an important emerging field. As this tutorial cannot be comprehensive, recent reviews have given a detailed overview.^13,14,19,20^

## 2. Mechanistic insights

**Fig. 2:**
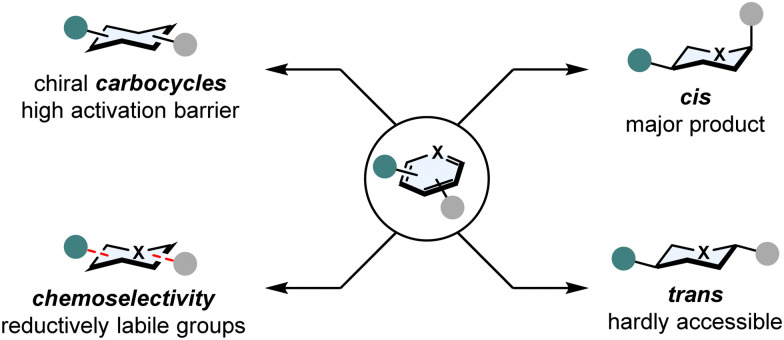
Major challenges in asymmetric arene hydrogenation: high kinetic activation barrier, stereoselectivity and chemoselectivity.

In spite of these advantages, four major challenges involving arene hydrogenation still exist (Fig. 2):^21^

### (1) Breaking aromaticity

In comparison to normal C–C or C–X double bonds, (hetero)arenes are additionally stabilised by aromaticity. The aromatic stabilisation energy can be as high as 36 kcal mol^−1^ for benzene^22^ and poses a significant kinetic barrier that needs to be overcome.

### (2) Catalyst poisoning

Heteroatoms such as sulphur or nitrogen are Lewis basic and tend to coordinate strongly to transition metals. This can result in a total shutdown in reactivity, the catalyst is poisoned.

### (3) Stereoselectivity

Hydrogenation of multi-substituted (hetero)arenes can form a plethora of diastereomers. While the all-*cis* configurated product is generally favoured according to the Horiuti-Polanyi mechanism,^23^ obtaining *trans* configurated products is possible by π-facial exchange,^24^ but still remains underdeveloped. Setting stereocentres remains a laborious task as well, since the discrimination of the two enantiotopic faces of a (hetero)arene is not trivial without and even with a directing group.

### (4) Chemoselectivity

Competing side reactions in the hydrogenation of (hetero)arenes possessing other reductively labile groups (*i.e.* nitriles, ketones, C–X double bonds or halogens) also create the problem of overreduction or hydrodefunctionalisation.

Three main strategies have been developed to overcome these obstacles: catalyst activation, substrate activation and relay catalysis. Catalysts can be activated by introducing additives that tune the steric and/or electronic environment of the catalyst. Primarily, the development of new ligands influenced the success of enantioselective arene hydrogenation.^13,14,25–28^ Moreover, it was often observed that the addition of iodine drastically increased the activity and enantioselectivity of metal complexes in hydrogenation reaction. It is proposed that iodine prevents the formation of inactive dinuclear metal complexes, thus increasing the catalytic rate.^29–31^ Substrates can be activated by installing protecting groups to the heteroatoms of the arene that could serve as a directing group for the catalyst and/or protect the catalyst from being poisoned.^32–35^ Also protonation of N-heteroarenes is a common strategy to lower the aromaticity of substrates.^36^ In relay catalysis, the aromaticity of a substrate is broken by an achiral catalyst, that however hydrogenates the substrate only partially. Then, a chiral catalyst sets the stereocentre to yield the final product.^37^

Various catalyst systems have been developed since the first reports in the late 90s. Most of these systems follow two major approaches to set the stereocentres, either homogeneous enantioselective or asymmetric heterogeneous hydrogenation. The following sections will give a deeper insight into both areas, discussing the advantages and disadvantages of these methods.

## 3. Enantioselective homogeneous hydrogenation

**Fig. 3:**
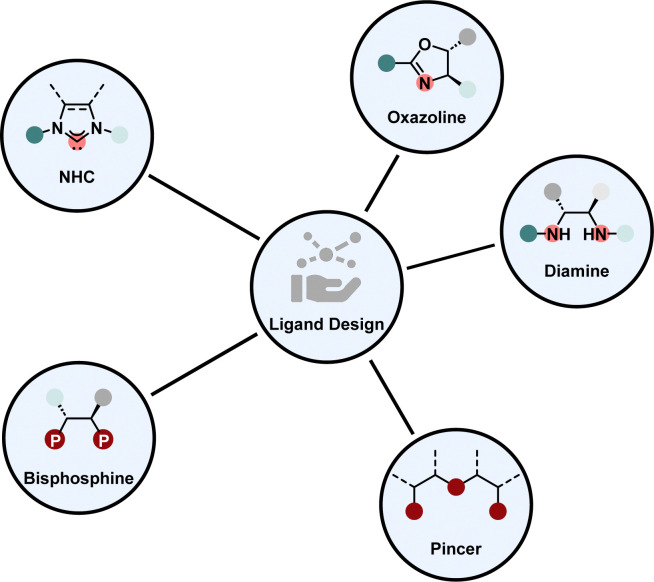
Prominent ligand motifs in asymmetric arene hydrogenation.

Enantioselective homogeneous hydrogenation is from a strategic perspective the most straight-forward way to obtain chiral saturated products. Homogeneous transition metal catalysts offer an inexhaustible amount of structural design possibilities. In praxis, there are only several prominent ligand types in combination with mostly a few noble metals, including iridium and ruthenium, which form highly selective and active catalysts to promote hydrogenation for a variety of different substrate motifs, we refer to them as privileged ligand motifs (Fig. 3). Each of these motifs possesses unique properties that make them invaluable for transition metal catalysis. For example, phosphines and NHCs are strong electron donating ligands influencing the electronic properties of the metal complex greatly.^38,39^ Diamines and oxazolines are synthesised from a broad chiral pool rendering them cheap and versatile.^40,41^ Pincer ligands coordinate with three atoms to the metal, therefore providing a high degree of stability to the metal complex. Hence, pincer ligands are often employed for low-valent, low-coordinate 3d-metal complexes.^42,43^ Additionally, each ligand type offers the possibility to be tuned electronically or sterically by changing the substitution pattern of the backbone.

One requirement for these catalysts is to remain stable under reductive reaction conditions. Depending on the external conditions, homogeneous metal complexes can form heterogeneous metal nanoparticles or clusters that can lead to a competing racemic hydrogenation reaction.^44–46^ For stereoinduction, the chiral environment at the metal centre needs to distinguish between two enantiotopic sides of the flat (hetero)arenes. The selectivity is often highly dependent on the solvent, temperature, additives and counterions present in the reaction medium. So far, many enantioselective hydrogenations for different (hetero)arenes including nitrogen-, oxygen-, sulphur-containing and carbocyclic arenes were published.

Most enantioselective arene hydrogenations target bicyclic heteroarenes and partially hydrogenate one ring. The aromatic stabilisation of bicyclic arenes is in general lower compared to two monocyclic arenes. In substrates with one heterocyclic ring and an annulated benzene, the latter one preferably remains intact because of the high stabilisation energy of benzene.

### 3.1. Quinolines

**Fig. 4:**
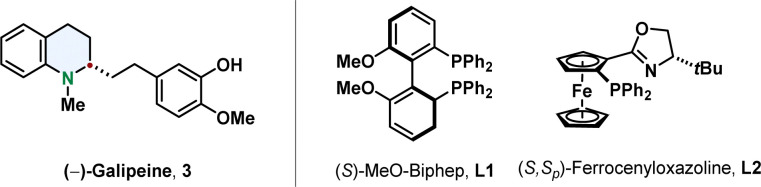
The alkaloid (–)-Galipine (left) containing a tetrahydroquinoline was accessed *via* enantioselective hydrogenation by Zhou and co-workers.^51^ The two ligands enabling high selectivities in asymmetric quinoline hydrogenation by Zhou and co-workers are shown on the right.^49,50^

With respect to the available methods already published, quinolines are the most facile substrates. The most common product of a quinoline hydrogenation is a 1,2,3,4-tetrahydroquinoline in which the nitrogen-containing ring is reduced and the more stabilised benzene ring is conserved.^47,48^ Efficient synthetic methods for their enantioselective hydrogenation go back to the beginning of the century when Zhou reported the iridium catalysed hydrogenation of 2,6-substituted quinolines with the phosphine ligand MeO-Biphep L1 or the ferrocenyloxazoline *N*,*P*-ligand L2.^49,50^ They proofed the strength of this new synthetic pathway by synthesising tetrahydroquinoline alkaloids like (–)-galipeine 3 in high enantiomeric excess (Fig. 4).^51^

**Scheme 1:**
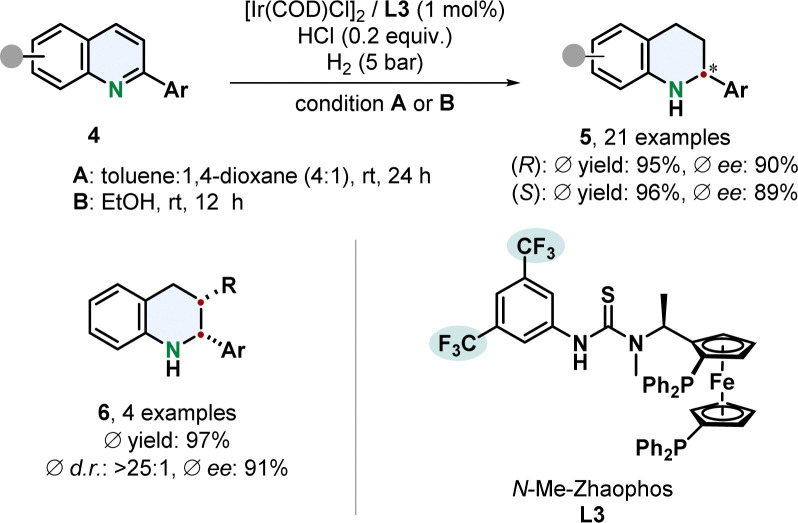
Enantiodivergent hydrogenation of 2-arylated quinolines by Dong, Zhang and co-workers.^52^

A remarkable enantiodivergent hydrogenation of 2-aryl substituted quinolines 4 was recently published by Dong, Zhang and co-workers (Scheme 1).^52^ An iridium catalyst with a thiourea based *N*-Me-Zhaophos ligand L3 facilitated efficient hydrogenation under acidic conditions.

Simple alteration of the solvent enabled access to both tetrahydroquinoline enantiomers 5 with only one stereoisomer of the catalyst. Highly polar solvents, especially protic solvents, gave the (*S*)-enantiomer with a TON of up to 1680 while less polar solvents switched the selectivity to the (*R*)-stereoisomer with a TON of up to 680. Deuterium labelling experiments indicate a difference in mechanism, that needs further investigation. Functionalised phenyl groups including halides, trifluoromethyl and dimethylamine as well as thiophene were tolerated well. 2,3-Disubstituted derivatives with an additional alkyl group yielded the *cis*-product 6 in high diastereoselectivity under slightly adjusted reaction conditions.

**Scheme 2:**
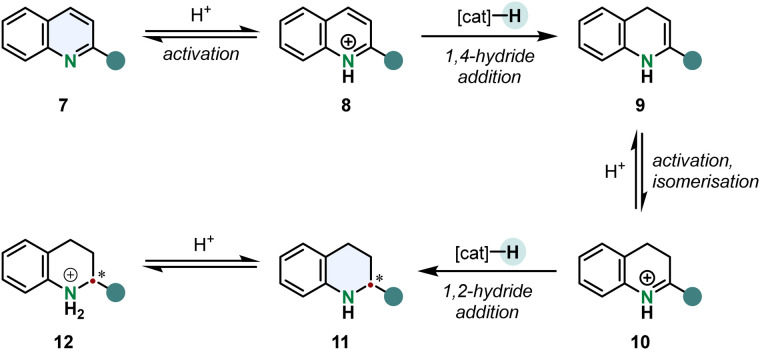
Outer sphere mechanism of the enantioselective hydrogenation of 2-substituted pyridines. After protonation the quinolinium cation undergoes a 1,4-hydride addition forming an enamine intermediate. Acid catalysed tautomerisation and subsequent 1,2-hydride addition gives the chiral tetrahydroquinoline cation.^52^

An arene hydrogenation is most of the times a multistep reaction involving different catalytic operations. A typical mechanism for the hydrogenation of quinolines is shown in Scheme 2. After substrate activation a 1,4-hydride addition gives the enamine 9 which can tautomerise to the imine 10. 1,2-Hydride addition to the activated iminium ion under stereocontrol of the catalyst yields the chiral tetrahydroquinoline 12 (THQ).^52^

**Scheme 3:**
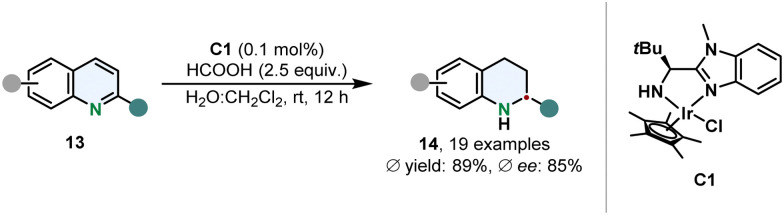
Transfer hydrogenation of 2-substituted quinolines with a water-soluble chiral iridium complex by Sun and co-workers.^53^

Sun and co-workers prepared a water stable iridium complex C1 with a simple *N*,*N*-ligand containing a benzimidazole scaffold for the transfer hydrogenation of 2-substituted quinolines 13 in an aqueous reaction medium under mild conditions (Scheme 3).^53^ Formic acid serves a dual role as hydrogen donor and to protonate C1 to increase its solubility. This catalytic hydrogenation appears to be appealing in particular because of its high TON of up to 33 000 and therefore low amounts of catalyst are required. Although 2-methylated quinolines could be hydrogenated in good ees, the enantioselective outcome proofed to be sensitive when switching to a different 2-substituent.

**Scheme 4:**
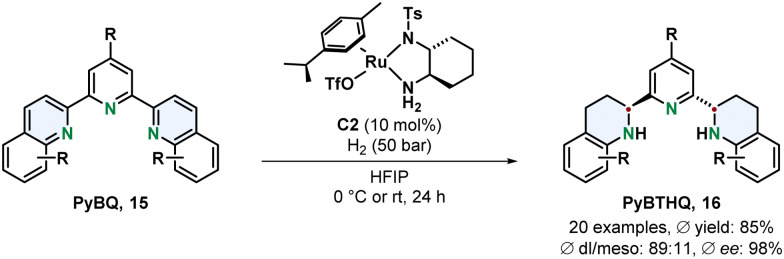
Synthesis of chiral terpyridine *N*,*N*,*N*-ligands *via* an enantioselective hydrogenation with a chiral Ru-diamine catalyst by Fan and co-workers.^55^

Chiral ruthenium diamine complexes are a privileged motive in asymmetric arene hydrogenation. A widely used example is the Ru-DPEN system introduced by Fan.^54^ More recently Fan and co-workers demonstrated the versatility of this catalyst class by using a chiral 1,2-diaminocyclohexane derived ligand for the efficient preparation of a new chiral terpyridine type class of *N*,*N*,*N*-ligands (Scheme 4).^55^ The tridentate Lewis basic substrates and resulting products make this transformation particularly challenging since such motives can strongly coordinate to the metal centre resulting in deactivation of the catalyst. The 2,6-bis(tetrahydroquinolin-2-yl)pyridines 16 (PyBTHQ) were obtained in high yields and excellent ees. These newly accessed PyBTHQ were successfully employed as ligands in a copper catalysed enantioselective Friedel–Crafts reaction.

**Scheme 5:**
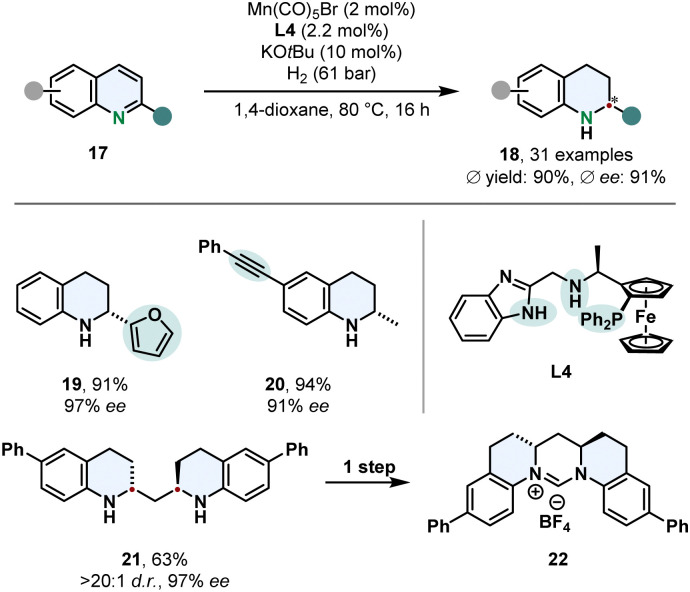
Manganese catalysed enantioselective hydrogenation of 2-substituted quinolines published by Lan, Liu and co-workers.^56^

The vast majority of asymmetric arene hydrogenations requires the use of precious and rare metals. Thus, switching to readily available and inexpensive 3d-metals is a more sustainable and highly desired method, but yet scarcely presented. A very remarkable example for its utility was recently published by Lan, Liu and co-workers who employed a manganese catalyst for the enantioselective hydrogenation of quinolines 17 (Scheme 5).^56^ By strategic alteration of the *N*,*N*,*P*-pincer ligand L4 they investigated the role of the benzimidazole scaffold and nitrogen substitution pattern. Free N–H groups in the linker and the imidazole proofed to be crucial for the reactivity and selectivity of the complex. DFT calculations of the transition state in the stereo determining step reveal the attractive π–π-interaction of the substrate with the benzimidazole favours one enantiomer. This synthetic method features an outstanding chemoselectivity for reductively labile groups. Tri- and disubstituted alkenes and alkynes are tolerated well. Low catalyst loadings were demonstrated on a gram scale with a TON up to 3840. An interesting application is the enantio- and diastereoselective hydrogenation of bis(quinoline-2-yl)methanes 21 followed by a simple condensation to access enantioenriched six membered NHC-prescursors 22.

### 3.2. Cascade reactions

**Scheme 6:**
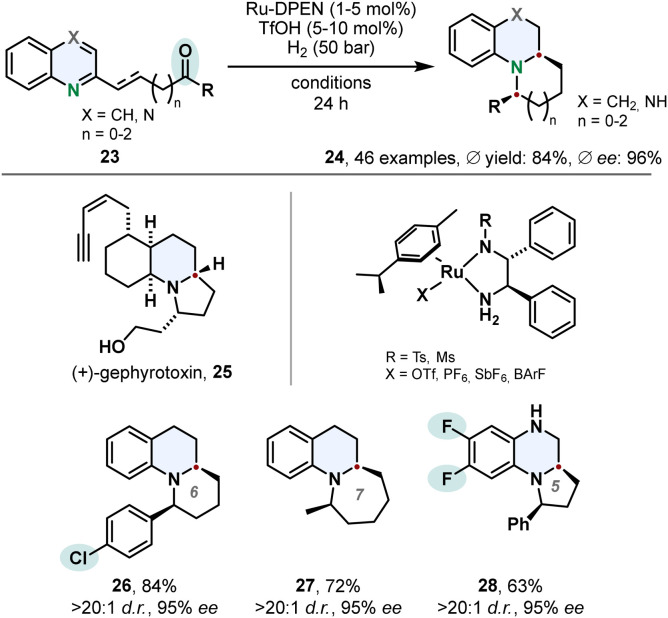
Cascade hydrogenation-condensation strategy for the synthesis of chiral indolizidines and quinolizidines involving an enantioselective hydrogenation published by Fan and co-workers.^57^

The hydrogenation of quinolines is not limited to the saturated bicyclic product class of tetrahydroquinolines. Suitable substrates containing a ketone moiety tethered to the 2-position can be utilised in a cascade hydrogenation-condensation sequence to access benzofused tricylic motifs 24. Fan and co-workers accessed chiral substituted indolizidines and quinolizidines in high diastereo- and enantioselectivities using Ru-DPEN catalysts and triflic acid (Scheme 6).^57^ Vital for this strategy is a balance of reactivity and selectivity. The quinoline moiety needs to be reduced to the cyclic amine in presence of the ketone which undergoes a subsequent intramolecular condensation to the corresponding imine. Also challenging is the following asymmetric reduction of the imine. Substrate activation with triflic acid was beneficial for the chemo- and enantioselectivity. Mechanistic studies revealed the second stereocentre is controlled by the configuration of the first stereocentre formed in the enantioselective hydrogenation of the quinoline core. The authors applied this synthetic method successfully on a gram scale in a formal total synthesis of the alkaloid (+)-gephyrotoxin 25.

**Scheme 7:**
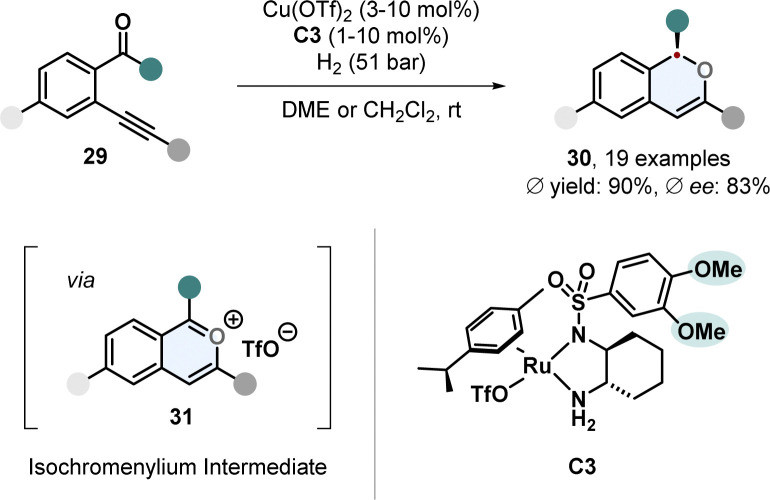
*In situ* formation of aromatic isochromenylium cations followed by enantioselective hydrogenation for the synthesis of chiral 1*H*-isochromenes by Yu, Fan and co-workers.^60^

One-pot cascade procedures are an emerging synthetic strategy.^58,59^ Instead of conducting a hydrogenation with a certain arene directly, the substrate is formed *in situ* by a sequence of reactions and then hydrogenated. This strategy is especially useful when the starting materials to form the arene are cheap and easily available. A tandem strategy involving two catalysts was used by Yu, Fan and co-workers to prepare chiral 1*H*-isochromenes 30 (Scheme 7).^60^*Ortho*-(alkynyl)arylketones 29 were used to generate reactive isochromenylium cations 31 by copper catalysis *in situ*, which were hydrogenated with a Ru-DPEN catalyst C3. It was further shown that the stereocentre of the 1*H*-isochromenes 30 can be used to induce stereoselectivity in a subsequent heterogenous hydrogenation to access the *cis*-configurated 1,3-substituted isochromanes.

### 3.3. Isoquinolines

**Scheme 8:**
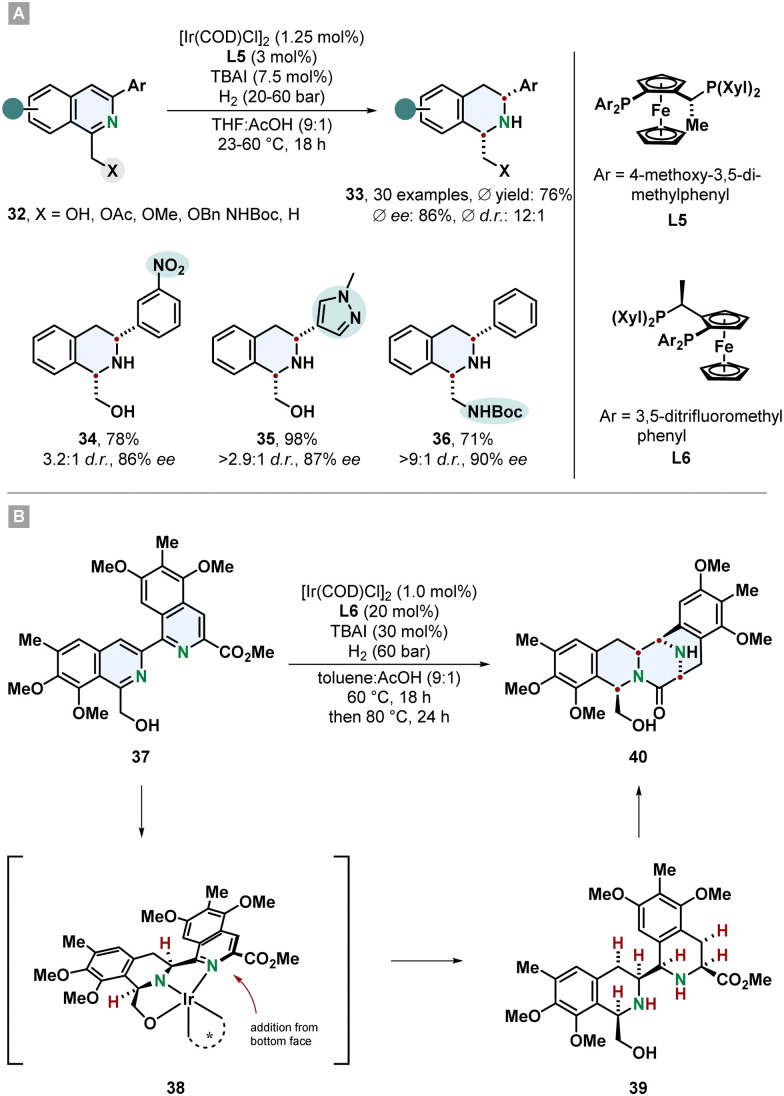
(A) Synthesis of chiral *cis*-configurated 1,3-disubstituted tetrahydroisoquinolines *via* enantioselective hydrogenation published by Stoltz and co-workers.^62^ (B) Application as key step in the total syntheses of the natural products (−)-jorunnamycin A and (−)-jorumycin by Stoltz and co-workers.^17^

Tetrahydroisoquinolines (THIQs) are an important class of partially saturated N-heterocycles and a prominent motif in many natural products and pharmaceutically active compounds. Synthetically, they can be made by electrophilic aromatic substitution reactions from substituted benzenes or *via* hydrogenation from the corresponding isoquinolines. Even though isoquinolines are closely related to quinolines, hydrogenation of them is more challenging and less selective methods and mild procedures are available. This might be associated to the lower reactivity of the substrate and the tendency of the Lewis basic THIQs to poison the catalyst.^61^ Particularly, highly substituted THIQs and those with Lewis basic functional groups are difficult substrates. A new method which tackles these problems was described by Stoltz and co-workers. 1,3-Disubstituted isoquinolines 32 containing a directing group in 1-position and an aryl substituent in 3-position were hydrogenated to the *cis*-configurated THIQs 33 in good yields, diastereo- and enantioselectivity (Scheme 8A).^62^ With an electronically optimized JosiPhos-ligand L5 and an iridium precursor a broad range of various isoquinolines including sterically hindered, heteroarene-substituted and with sensitive functional groups reacted under mild conditions without the need for a strong acid or formation of the isoquinolinium salt for substrate activation. Reduction labile nitro- and nitrile-substituted aryl groups were unharmed. Besides the predominantly used hydroxymethyl-directing group also different ethers, acetate and a protected amine were sufficient. With a non-directing methyl group, the corresponding THIQ was still obtained in high stereoselectivity. Electron poor isoquinolines with a 7-fluoro group worked smoothly. This is of synthetic interest because THIQs with electron withdrawing groups are difficult to access with common Pictet-Spengler reactions. For many substrates 20 bar of hydrogen pressure and room temperature were proficient conditions. It is noteworthy that instead of THF the green alternative 2-methyl-THF can be used as replacement with only a slight decrease in selectivity. The obtained *β*-amino alcohol containing THIQs could successfully be utilised to build up more complex chiral ring-structures like an oxazolidine- and oxazolidone-fused THIQ with a 6,6,5-tricycylic skeleton, a morpholinone 6,6,6-tricycle and even a pentacyclic scaffold.

An impressive example for the synthetic power of asymmetric arene hydrogenation was shown by Stoltz and co-workers in the total syntheses of the complex natural products (−)-jorunnamycin A and (−)-jorumycin (Scheme 8B).^17^ In a key step of their route the bis-isoquinoline 37 was hydrogenated creating four new stereocentres under excellent control of diastereo- and enantioselectivity in a single step and setting the stage for the following lactam condensation to form the highly substituted pentacyclic scaffold 40. Similar to the above described method an iridium catalyst with a modified JosiPhos ligand L6 was used. This non-biomimetic approach enabled the synthesis of more electron rich partially deoxygenated derivatives for the strategic investigation of the location of active sites for the cytotoxicity.

Arene hydrogenation reactions are mainly *cis*-selective because the hydrogen is transferred from one catalytic face. *trans*-Selective arene hydrogenations are very rare and until recently no example for an asymmetric version was known.^63^ Mechanistically a π-facial exchange in between the stepwise hydrogenation is required.

**Scheme 9:**
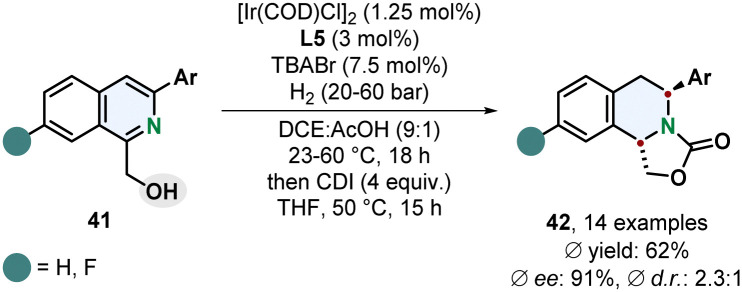
*trans*-Selective directed enantioselective hydrogenation of 1,3-disubstituted isoquinolines with subsequent formation of an oxazolidine-2-one by Stoltz and co-workers.^63^

Stoltz and co-workers recently reported that the diastereoselectivity for the enantioselective hydrogenation of 1,3-disubstituted isoquinolines 41 can be switched to a *trans*-selective method when the coordinating solvent THF was exchanged for the non-coordinating solvent DCE (Scheme 9).^63^ The right choice of additive turned out to be crucial for the selectivity. TBABr and TBACl provided *trans*-selectivity whereas TBAI led to a favoured formation of the *cis*-diastereomer. Although the *trans*-selectivity for the chloride donor was slightly better the enantioselectivity was diminished compared to the bromide donor. This method requires a 1-hydroxymethyl group as coordinating group limiting the scope of accessible products. Other coordinating groups could not switch the selectivity in favour of the *trans*-product. In general, the selectivity for the *trans*-diastereomer is certainly lower than in the *cis*-selective reaction, still the enantioselectivity is high and the ability to access both chiral diastereomers with the same catalyst by simply switching the reaction medium and additive is synthetically a very useful methodology.

### 3.4. Five-membered heterocycles and fused rings

**Scheme 10:**
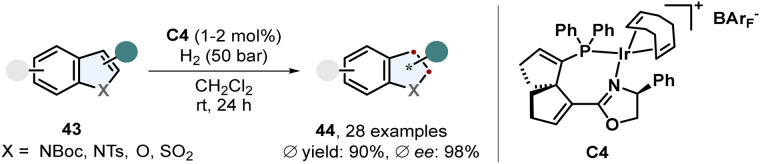
Iridium catalysed enantioselective hydrogenation of 2- and 3-substituted indoles and benzofurans by Han, Ding and co-workers.^64^

Benz-annulated saturated five membered heterocycles display a diverse class of different scaffolds. Many natural products and pharmaceutically active compounds contain such chiral building blocks including indolines and 2,3-dihydrobenzofurans. These motifs are readily accessible *via* enantioselective arene hydrogenation. A recent example of a very efficient procedure was published by Han, Ding and co-workers (Scheme 10).^64^ Protected 2- and 3-substituted indoles 43 were converted to the corresponding indolines 44 in excellent enantioselectivity and yields using a spirocyclic SpinPHOX-iridium complex C4. Halide substituents on the benzene ring were well tolerated and it was shown that this catalyst also works for simple benzofurans.

**Fig. 5:**
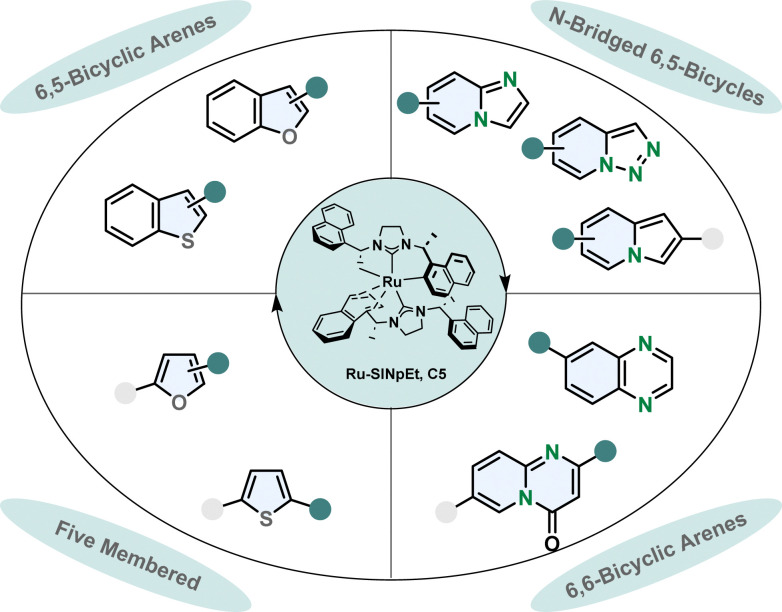
Overview of the scope of diverse heterocycles for the efficient enantioselective hydrogenation with the privileged Ru-SINpEt catalyst by Glorius and co-workers.^65^

Designing a catalytic system which is capable of performing asymmetric hydrogenations of various aromatic scaffolds in high stereoselectivity while maintaining a broad functional group tolerance is a challenging task. The ruthenium-SINpEt catalyst reported by Glorius and co-workers proved to be exceptionally versatile for the asymmetric hydrogenation of multiple heterocycles (Fig. 5).^65^ Different frameworks including five membered monocycles, 6,5-bicycles and 6,6-bicycles and different heteroatoms namely oxygen, nitrogen and even sulphur gave reliably good selectivity and provided efficient hydrogenation. The heteroarenes can be electronically rich or poor, with a single or multiple heteroatom(s) in various positions including nitrogen bridged scaffolds.^66–71^ The range of substrates is presented in more detail in a previous review.^65^

In most enantioselective hydrogenation reactions of polycyclic heteroarenes the ring containing the heteroatom(s) is the one that is hydrogenated more readily. Carbocyclic arenes usually have higher aromaticity and are less polarised compared to heteroarenes.^48^ However, with the Ru-SINpEt system Glorius and co-workers for the first time were able to selectively hydrogenate the carbocylic benzene ring of quinoxalines enantioselectively with retention of the pyrazine motif.^72^

### 3.5. Hydrogenation of carbocylic arenes

**Fig. 6:**
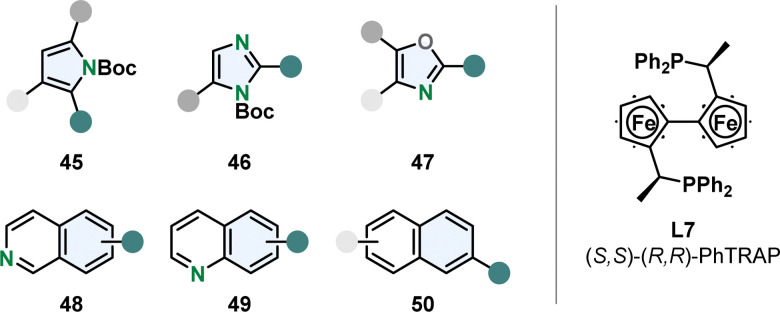
Overview of the suitable (hetero)arenes for the efficient enantioselective hydrogenation with the privileged Ru-PhTRAP catalyst established by Kuwano and co-workers.^47,73–76^

Chemoselective hydrogenation of the more stabilised carbocyclic ring is challenging and therefore rare. Kuwano and co-workers were able to perform enantioselective hydrogenation of the benzene moiety of quinolines 48 and isoquinolines 49 with a Ru-PhTrap catalyst (Fig. 6).^47,73^ According to the authors the combination of the *trans*-chelating PhTRAP ligand L7 with a large bite angle, polar solvents and alkali carbonate as base contributes to the rare chemoselectivity. The Ru-PhTRAP is a privileged catalyst for arene hydrogenation and was in previous works successfully utilised for the hydrogenation of various five membered monocycles including multisubstituted oxazoles 47 and protected imidazoles 46 and pyrroles 45 (Fig. 6).^74,75^ They also gave the first example for the enantioselective partial hydrogenation of naphthalenes 50 using the same catalyst (Fig. 6).^76^ A few examples of symmetrical 2,6- and 2,7-disubstituted naphthalenes were hydrogenated in good yields and selectivity to the corresponding tetralins. However, this method is limited to specific substituents and substitution patterns. Alkoxy- or ester-groups were required, dialkylated naphthalenes resulted in no reaction possibly due to the lack of the oxygen lone pair to coordinate to the catalyst. In unsymmetrical substrates with a 2-ethoxy group and alkyl or aryl substitution in 6-position, the ring containing the coordinating group was hydrogenated in moderate to good regio- and high enantioselectivity.

**Scheme 11:**
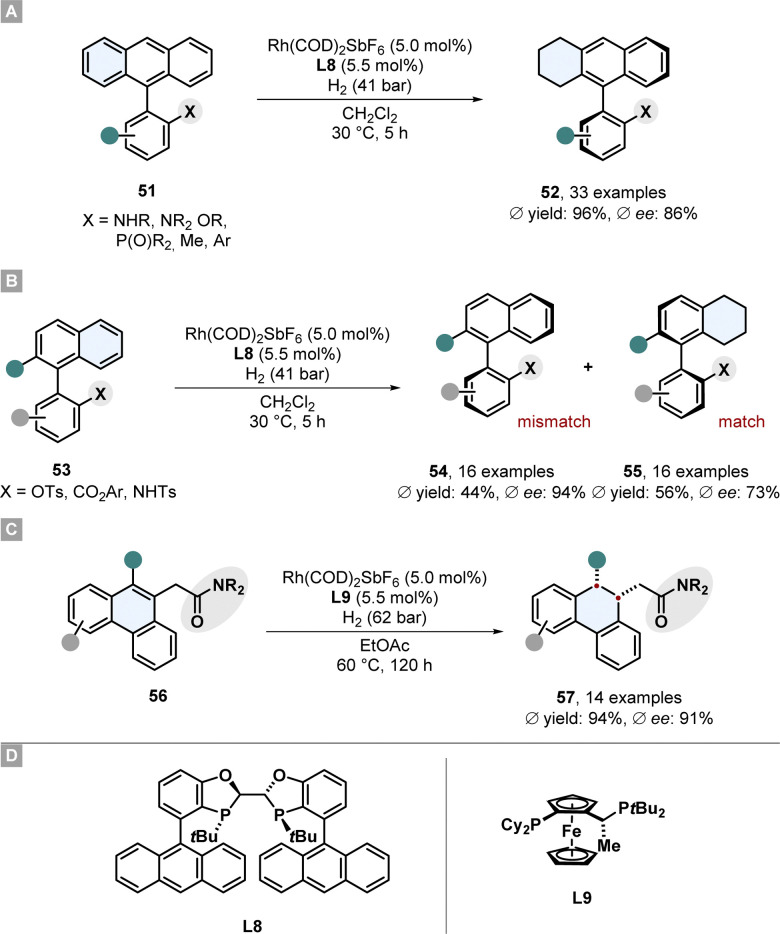
Asymmetric hydrogenation of carbocylic motifs to access axial-chiral and central-chiral products by Zhou and co-workers.^77^ (A) Desymmetrisation of 9-phenyl substituted anthracenes. (B) Kinetic resolution of phenyl-substituted naphtalenes. (C) Enantioselective hydrogenation of 9-subtituted phenanthracenes. (D) Structures of the ligands used for these reported hydrogenations.

Recently Zhou and co-workers reported the asymmetric partial hydrogenation of different polycyclic all-carbon arenes to access axially chiral product motifs with a rhodium-phosphine catalytic system (Scheme 11).^77^ A broad scope of 9-phenyl substituted anthracenes 51 bearing a protected amine or ether directing group were desymmetrised and also two examples without a directing group were shown (Scheme 11A).

Crucial for the enantioselectivity is the strongly electron donating and sterically very demanding WingPhos ligand L8, the choice of counterion of the rhodium salt and the solvent. A weakly coordinating SbF_6_^−^ anion and non-coordinating dichloromethane proved to be beneficial. The utility of this desymmetrisation was shown for the synthesis of axial chiral phosphine ligands by hydrogenating the corresponding phosphine oxide substrates followed by reduction to the phosphines. This procedure was successfully applied for the kinetic resolution of racemates of phenyl-substituted naphthalenes 53 (Scheme 11B). The matching atropisomer was partially hydrogenated at the naphthalene because of its lower aromaticity per ring. Both the enantioenriched mismatched starting material 54 and the partially hydrogenated product 55 were obtained in good selectivity. Moreover, phenanthracenes 56 bearing a coordinating amide in 9-position were enantioselectively hydrogenated at the central ring to yield central-chiral partially saturated products 57 in high selectivity and good yields (Scheme 11C). For this hydrogenation a JosiPhos ligand L9 was found to be the best and the reaction conditions were adjusted. Its noteworthy to comment that although also 9,10-disubstituted motifs gave high ees, the necessity of the directing group and a scope which is limited to non- or methyl-substituted external rings limits its applicability.^77^ In a previously reported similar method by Zhou and co-workers 9-acetamide substituted phenanthracenes could also be hydrogenated in high selectivity.^78^

**Scheme 12:**
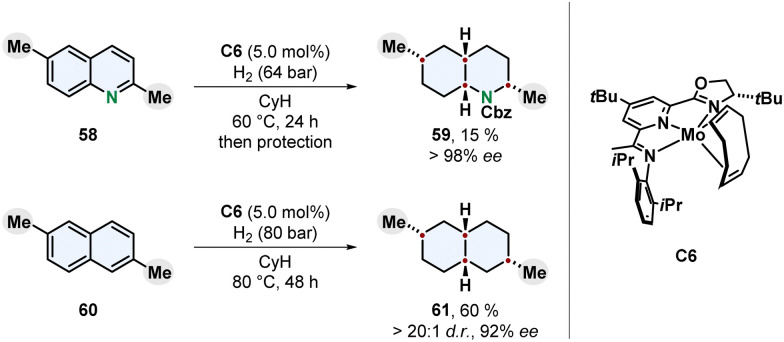
Molybdenum catalysed enantioselective full hydrogenation of quinolines and naphthalenes to the corresponding decahydroquinolines and decalines by Chirik and co-workers.^79^

An intriguing new catalyst design for the enantioselective and diastereoselective complete hydrogenation of quinolines and naphthalenes was recently published by Chirik and co-workers (Scheme 12).^79^ Earth-abundant molybdenum was chosen as more sustainable transition metal and strategic evaluation of ligands led to an oxazoline imino(pyridine) motif. A bulky 4-*tert*-butyl group on the pyridine ring turned out to be crucial for high enantioselectivity, although facing away from the metal centre. The added steric repulsion might prevent hydrogenation of the pyridine and finally erosion of the catalyst leading to racemic background reactions lowering the ee.

Nitrogen-containing and carbocyclic fused bicycles were investigated as substrates. Similar to quinolines the hydrogenation of naphthalenes usually yields tetralins because a highly stabilised benzene ring is conserved.^48^ 2,6-Dialkylated naphthalenes provided the fully reduced *cis*-configurated decalines in high enantioselecitivity. For the 1,7-dimethylated naphthalene 60 the *trans*-product 61 was observed as major diastereomer. Different substitution patterns including 1,8-disubstituted and monosubstituted naphthalenes resulted in reduced selectivity, although the monosubstituted 2-methyl derivative gave a moderate ee. Interestingly, while 2,6-disubstituted quinolines were hydrogenated to the corresponding decahydroquinolines, monosubstituted quinolines, isoquinolines, quinoxalines and 2,8-disubstituted quinolines only yielded mixtures of partially saturated products. The chemoselectivity for whether the carbocyclic ring or the nitrogen containing ring was hydrogenated was found out to be highly dependent on the steric environment and location of the substituent. The enantioselectivity on the other side, was also high for 3- and 4-substituted quinolines. Mechanistic investigations support a mode of enantioinduction based on buried volume without a coordination of the nitrogen to the catalyst. The development of a molybdenum-based enantioselective catalyst for arene hydrogenation with unique reactivity marks a progress towards sustainability. However, relatively high catalyst loadings, low yields for heteroarenes and a scope restricted to alkyl substituents limit the applicability of this new system and demand further studies.^79^

### 3.6. Six-membered N-heterocycles

**Fig. 7:**
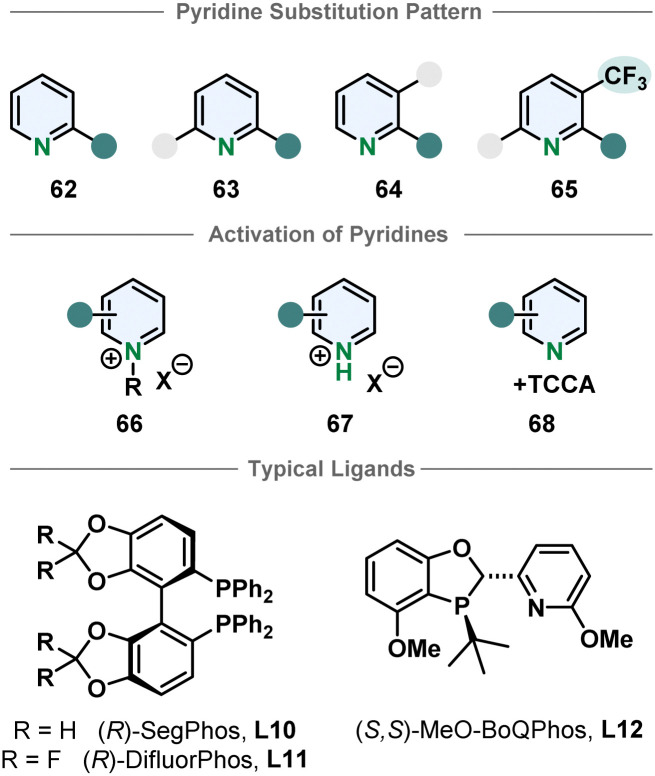
Top: Overview of the susceptible pyridine motifs for enantioselective hydrogenation.^80–90^ Middle: Common activation strategies of pyridines for homogenous asymmetric hydrogenation. Bottom: Examples for successfully used ligands in enantioselective pyridine hydrogenation.

Compared to their bicyclic counterparts, there are fewer synthetic methods for the enantioselective hydrogenation of six membered monocyclic heteroarenes. Pyridines in particular are very challenging substrates because of the high aromatic stabilisation and Lewis basicity of the piperidine products, deactivating the catalyst. Therefore, most methods require activation by derivatisation to a pyridinium salt. Benzylated pyridinium-salts can be hydrogenated enantioselectively with various iridium phosphine complexes. Most of these procedures only allow substitution in 2-position.^80–85^ Zhang and Chen more recently used an iridium-SegPhos catalytic system to hydrogenate 2-aryl-3-phtalimidopyridinium salts to the resulting *cis*-configurated chiral piperidines in high selectivities.^86^ The pyridinium salts formed with strong Brønsted acids are also typical substrates. Mashima and co-workers hydrogenated 2,6- and 2,3-substituted pyridinium halides asymmetrically.^87,88^ Zhou and co-workers were also able to use 2,3,6-trisubstituted pyridinium salts when an electron withdrawing trifluoromethyl group was located in 3-position.^89^ Pyridines could also be activated *in situ* with TCCA to yield chiral piperidines.^90^ Bao, Zhang and co-workers used an enantioselective pyridine hydrogenation of cyclic pyridinium salts to obtain chiral indolzidines. The cyclic pyridinium salts can be synthesised form 2-(2-acylphenyl)-pyridines in two steps. Even though the enantioselectivity of the hydrogenation is moderate, it is a noteworthy approach to access a different product class.^91^ Despite these advances, enantioselective pyridine hydrogenation is still mostly restricted to limited substitution patterns, low functional group tolerance, especially of groups directly attached to the pyridine core, and harsh methods necessary for substrate activation (Fig. 7).

Chiral piperazines are common scaffolds in pharmaceutically active compounds. From a strategic perspective the most straight-forward way to access them is a single-step enantioselective hydrogenation. Still, there are only few reports using this pathway.^92^ Compared to piperidines the piperazines contain an additional amine that can lead to catalyst poisoning. Zhou and co-workers developed methods to hydrogenate 3-monosubstituted, 2,3- and 3,5-disubstituted pyrazines with iridium-phosphine catalysts in good selectivities after activation by benzylation to the corresponding pyrazinium salt.^93^

The choice of counterion turned out to be crucial for the selectivity and depending on the substitution pattern different ligands and solvent systems were used. The authors demonstrated the utility of their transformation by synthesising the NK1 receptor antagonist Vestipitant 77 from the benzylated piperazinium in three steps. Zhou, Shi and co-workers recently reported the enantioselective hydrogenation of 5,6-arylated pyrazin-2-ols 72 using a palladium-TolBINAP catalyst.^94^ The enantioselectivity is high but the method is mainly limited to substrates with identical 5- and 6-substituents.

**Fig. 8:**
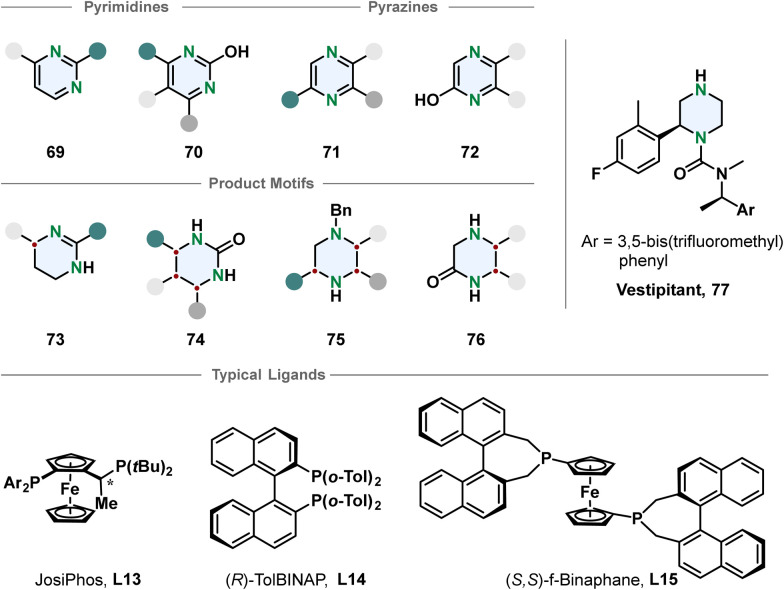
Top: Overview of pyrimidines and pyrazines successfully employed in an enantioselective hydrogenation reaction.^93–97^ Middle: Corresponding product motifs after hydrogenation. Bottom: Examples for successfully used ligands in these hydrogenation reactions.

Another important class of N-heterocycles are undoubtedly pyrimidines. Besides the above-mentioned challenges of overcoming aromatic stabilisation and formation of strongly coordinating products, the fully hydrogenated 1,3-diazinanes are rather sensitive to hydrolysis because of the cyclic aminal moiety. Partial enantioselective hydrogenation to the 1,4,5,6-tetrahydropyrimidines is feasible when a stabilising 2-substituent is introduced preventing the formation of an aminal. Kuwano and co-workers converted 2-arylated pyrimidines 69 to the corresponding cyclic amidines 73 with an iridium-JosiPhos catalyst.^95^ Substrate activation with iodine and ytterbium triflate promoted high enantioselectivity for a broad scope of 4-alkylated or arylated substrates 73. 2-Hydroxpyrimidines 70 are suitable substrates for the synthesis of chiral cyclic ureas 74*via* hydrogenation as shown by Shi, Zhou and co-workers.^96,97^ The 2-hydroxypyrimidines 70 have a lower aromaticity and exist in an equilibrium with the tautomeric pyrimidone. Activation with organic acids or TCCA enabled effective hydrogenation of mono- and multi-substituted 2-hydroxypyrimidines in overall good selectivities (Fig. 8).

## 4. Asymmetric heterogeneous hydrogenation

Although heterogeneous catalysts have significant advantages over homogeneous systems such as efficient recycling and minimisation of metal traces in the products, the field of asymmetric arene hydrogenation has been dominated by homogeneous catalyst systems. This might be due to the higher activity and stereoselectivity of homogeneous catalysts and the facile manipulation of electronic and steric properties by ligand design. Nonetheless, asymmetric heterogeneous systems have made significant advances in the past years and are applied in various processes.^98–101^

**Fig. 9:**
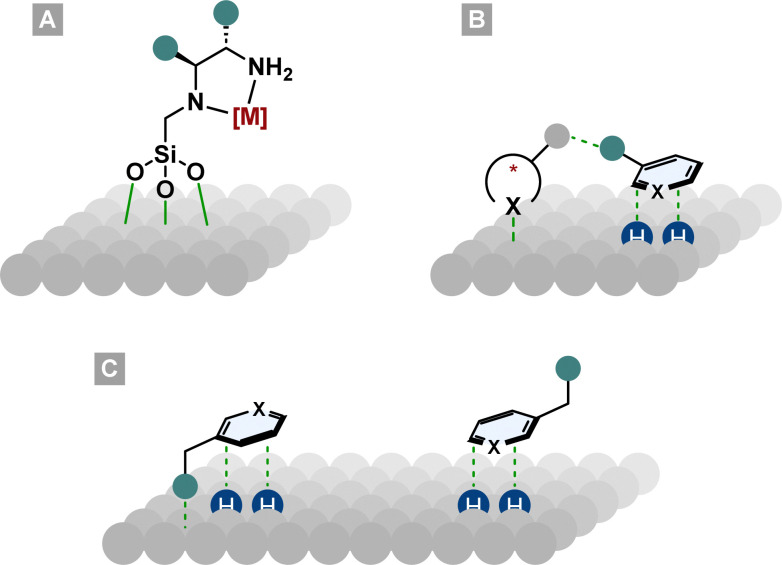
Asymmetric induction in heterogeneous catalysis. (A): Homogeneous catalyst bound to the metal surface. (B): Chiral modifier on the metal surface, interacting with the substrate. (C): Electronic attractive or steric repulsive interactions of a molecule with the metal surface.

The field of asymmetric heterogeneous hydrogenation can be divided into three subareas. First, immobilisation of privileged homogeneous catalyst systems on a surface. Therefore, chiral ligands or preformed metal complexes are bound covalently or non-covalently (*i.e.* ionic interactions) to a support such as silica (Fig. 9A).^102,103^ Second, hydrogenation over surfaces modified with chiral molecules. By the addition of chiral complexing molecules that can interact with the substrate, a chiral environment on the metal surface is created (Fig. 9B).^101^ The best known and studied modified surface is the Orito system. His group used cinchona alkaloids to modify a platinum surface for the enantioselective hydrogenation of pyruvates and other α-functionalised ketones.^104^ Despite their success in other areas, these two methods have produced only moderate to bad ees in the field of asymmetric arene hydrogenation. More promising results can be found in the third subarea, the diastereoselective hydrogenation of chiral molecules. These chiral substrates either have steric repulsive or electronic attractive interactions with the metal surface so that one diastereotopic face of the arene is preferentially hydrogenated (Fig. 9C).^105^

### 4.1. Chiral auxiliary assisted diastereoselective hydrogenation

**Scheme 13:**
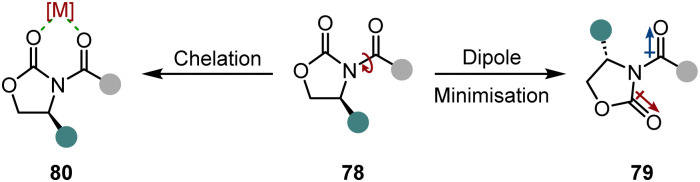
Conformational alignment of Evans’ auxiliary.

An easy and apparent approach to employ chirality to a molecule is the use of chiral auxiliaries. Ideally, they can be easily installed before and cleaved after the hydrogenation reaction, functioning as a traceless auxiliary. Also crucial for the success of the enantioinduction is the conformational rigidity of the auxiliary. Taking the Evans’ auxiliary 78 as an example, even though it is only bound to the substrate by a single bond, it preferentially adopts one of the two possible conformations due to dipole minimisation 79 or chelation 80 (Scheme 13). Hence, only one diastereotopic side of the substrate is shielded, yielding high diastereoselectivities.

**Scheme 14:**
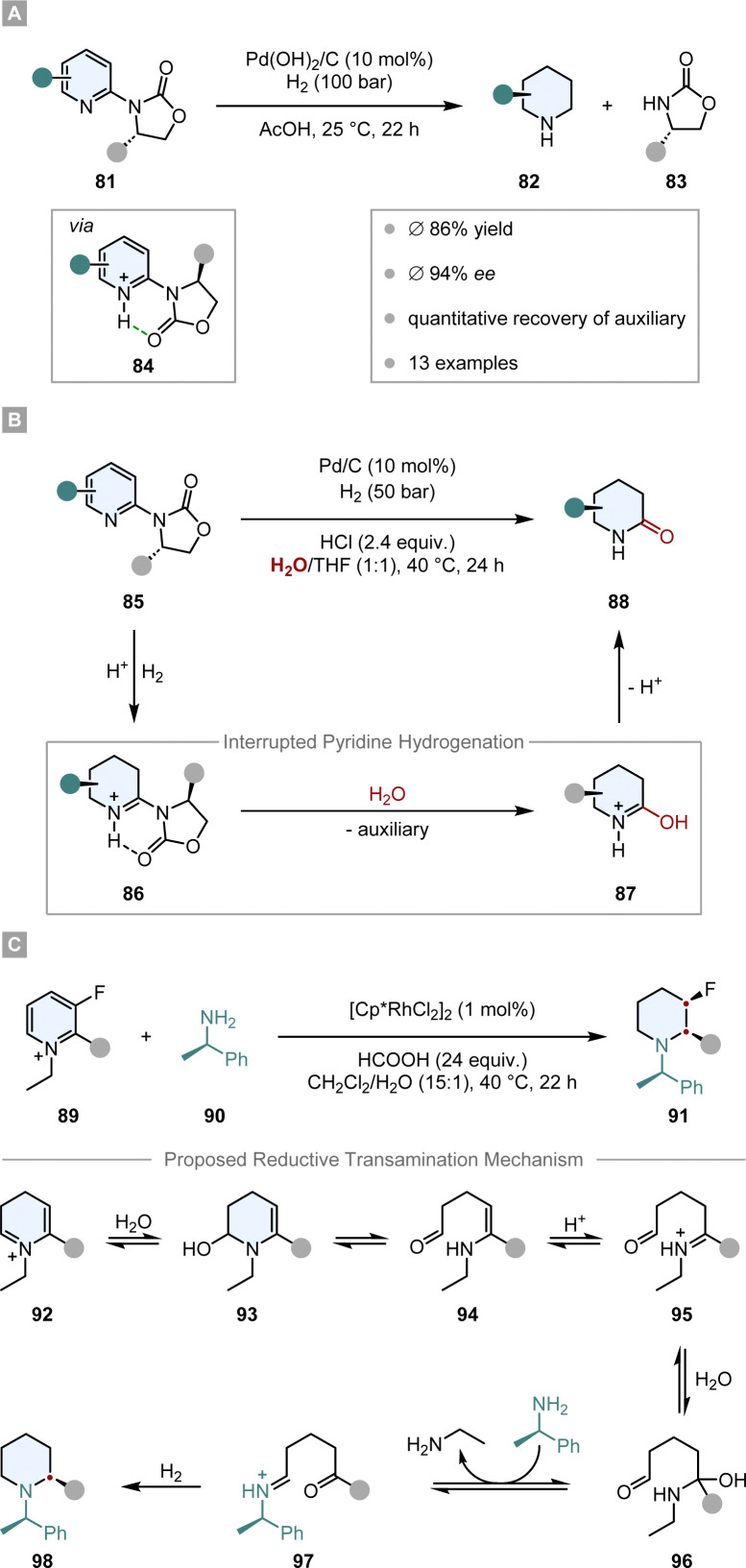
Chiral auxiliary assisted asymmetric hydrogenations of pyridines and pyridinium salts.^108,110,112^

First attempts of diastereoselective hydrogenation were performed by Lemaire in 1994. Employing menthoxyacetic acid as chiral auxiliary to *o*-cresol, he was able to achieve a promising but low 10% ee.^106^ Four years later Besson and co-workers used pyroglutamic acid methyl ester as a more rigid chiral auxiliary in the hydrogenation of *o*-toluic acid.^107^ A highly diastereoselective arene hydrogenation resulted, with an impressive de of 95% at 49% conversion. The Glorius group developed a diastereoselective pyridine hydrogenation using the Evans’ auxiliary as chiral unit and Pd(OH)_2_/C as achiral catalyst (Scheme 14A).^108^ Crucial for the success of the reaction is the acidic medium which serves three important functions. The protonation of the pyridine facilitates a hydride attack and lowers the kinetic barrier, additionally it negates the Lewis basicity of the free electron pair on the nitrogen, preventing it from poisoning the catalyst. More importantly, it is believed that a hydrogen bond between the protonated nitrogen and the carbonyl group locks the conformation of the auxiliary, assuring the efficient shielding of one diastereotopic face and consequently yielding high ees (*ϕ* 94% ee). With this method they were able to set up to three stereocentres in a single operation. Another aspect that renders this reaction environmentally benign is the recyclability of the auxiliary. Under the reaction conditions it is cleaved after hydrogenation – serving as a traceless auxiliary – and can be recovered and reused without any loss in enantioselectivity. This protocol found application in the enantioselective synthesis of (−)-isooncinotine.^109^

Recently, Glorius and co-workers disclosed an asymmetric interrupted pyridine hydrogenation which builds upon these results (Scheme 14B).^110^ During the hydrogenation cycle the imine intermediate 86 accumulates and can be intercepted by a nucleophile. By switching the solvent from AcOH to a mixture of THF/H_2_O they could utilise water as a nucleophile to attack the imine intermediate. The product 88, enantioenriched δ-lactams, could be synthesised in high ees (*ϕ* 90% ee) and yields (*ϕ* 72%) and it could be shown that this method can be applied in the synthesis of enantiopure δ-amino acids. Another application was demonstrated in the elegant total synthesis of (−)-senepodine F by Ishikawa and co-workers.^111^ As one of the key steps, one piperidine moiety was constructed by the asymmetric interrupted pyridine hydrogenation protocol reported by Glorius. The final product was obtained in 13% overall yield over 17 steps.

Another interrupted hydrogenation process making use of a chiral auxiliary is the recently published reductive amination of pyridinium salts reported by Xiao and co-workers (Scheme 14C).^112^ This protocol describes an asymmetric transfer hydrogenation employing [Cp*RhCl_2_]_2_ as achiral catalyst and formic acid as the hydrogen source. The authors discovered that if chiral amine 90 is added to the reaction mixture it is incorporated into the product, giving rise to enantiomerically pure piperidines 91. After intensive mechanistic investigations it is proposed that as a key step the dihydropyridinium intermediate 92 is intercepted by water, which leads to a ring opening and an expulsion of ethylamine. After condensation with chiral amine 90 and ring closure, a final diastereoselective hydrogenation of the imine leads to the enantiomerically pure piperidine 98. Remarkably, this protocol tolerates many reductively labile groups that are usually not stable under normal hydrogenation conditions. Easily reducible groups such as nitro, cyano or carbonyl groups as well as halogens, which are prone to hydrodefunctionalisation, are well tolerated. This underlines the mildness of the reaction by not employing high hydrogen pressure, but only transfer hydrogenation conditions. The products are usually obtained in high diastereoselectivities and yields.

Besides these recent examples, there has been also a few other highly diastereoselective (≥90% ee) hydrogenations of furanes,^113,114^ pyrroles^115^ and quinolines^116^ utilising chiral auxiliaries. Nonetheless, introducing chiral auxiliaries that possess enough conformational rigidity to shield one diastereotopic face efficiently is a challenging task. Additionally, the auxiliaries need to be readily synthesised from the chiral pool and the facile installation, cleavage and recyclability is essential for the protocol to be environmentally benign. These drawbacks limit the area of application for this strategy.

### 4.2. Substrate induced asymmetric diastereoselective hydrogenation

**Scheme 15:**
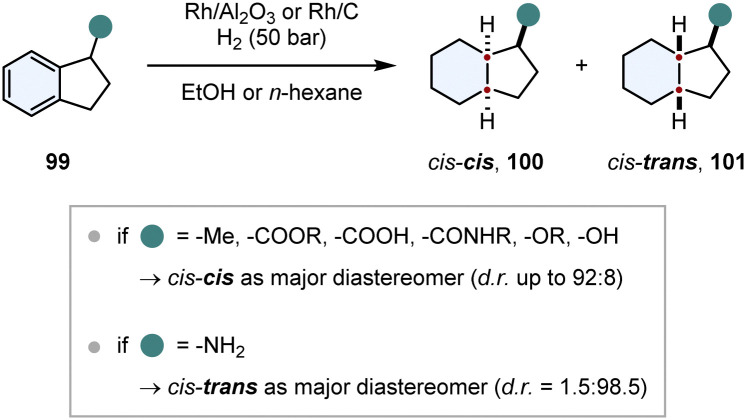
Investigation of substituent effects on the diastereoselective hydrogenation of 1-substituted indanes.^117,118^

In comparison to chiral auxiliaries, substrate induced diastereoselective hydrogenation makes use of already existing stereocentres in close proximity to the arene. This strategy needs to fulfill similar requirements for a good stereoinduction: the stereocentre should be close to the prochiral arene, its structure should be rigid and the chiral substituent should have a steric repulsive or an electronic attractive interaction with the catalyst, also called “haptophilicity”. A remarkable study about the substituent effects on diastereoselective hydrogenation was published by Prins and co-workers (Scheme 15).^117,118^ Because of the rigid ring structure and the proximity of the stereocentre, 1-substituted indanes 99 are suitable substrates to study these effects. If steric repulsive interactions dominate, then hydrogen is transferred to the opposite face of the substituent and the *cis–cis* product 100 is obtained as major diastereomer. Conversely, if the substituent is haptophilic, hydrogen is transferred to the same face of the substituent and the *cis–trans* product 101 is favoured (compare Fig. 9). For most of the substituents (ethers, alkanes, alcohols, amides, carboxylic acids and esters) steric repulsive effects were dominating and the *cis*–*cis* product 100 was obtained as the major diastereomer. Even slightly coordinating groups like an alcohol could only diminish the d.r. a little bit to 57 : 43. Only amino substituents exhibited a strong attractive interaction, reversing the d.r. completely to 1.5 : 98.5 in favour of the *cis–trans* product 101.

**Scheme 16:**
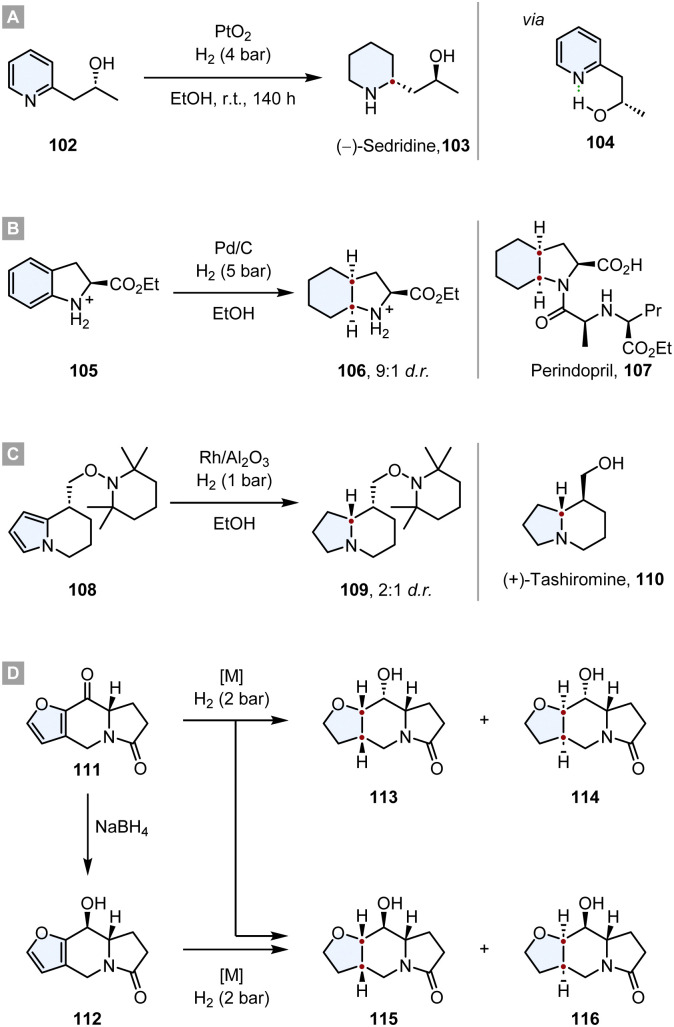
Substrate induced asymmetric diastereoselective hydrogenations in total syntheses of natural products and drug candidates.^119,120,122,123^

The value of this strategy was demonstrated multiple times in the syntheses of natural products and promising drug candidates. In 1968 Cooke and Fodor published the synthesis of (−)-sedridine 103.^119^ They took advantage of a hydroxyl group adjacent to the pyridine ring, inducing a high diastereoselectivity. By IR studies they discovered that the hydroxyl group is completely intramolecularly hydrogen bonded to the pyridine nitrogen. The authors propose that this leads to a rigid ring structure in which the methyl group shields one face of the ring, leading to a high d.r. (Scheme 16A).

In the synthesis of Perindopril 107, an inhibitor of Angiotensin Converting Enzyme (ACE), the optically pure octahydroindole core was constructed by a diastereoselective hydrogenation of the corresponding indoline 105, yielding the product 106 with a d.r. = 9 : 1.^120^ After converting 106 to the corresponding *tert*-butyl ester, the optical purity was determined as >98% ee. Further synthetic transformations yielded the desired drug (Scheme 16B).

Tashiromine is an indolizidine alkaloid, a group of natural products that can be extracted from various plants and animals with potent biological activity and pharmacological effects.^121^ Shortly after the first extraction of natural tashiromine, Branchaud and co-workers synthesised (+)- and (−)-tashiromine to address the stereochemistry question.^122^ After assembling the tetrahydroindolizine core 108*via* an enantioselective pyrrole/cobaloxime π-cation cyclisation, a diastereoselective hydrogenation with Rh/Al_2_O_3_ yielded the indolizidine 109 with a d.r. = 2 : 1. Further cleavage of the protecting group and epimerisation yielded (+)-tashiromine 110 (Scheme 16C).

Another interesting synthesis of an indolizidine *via* a diastereoselective hydrogenation was reported by Daïch and co-workers.^123^ Starting from the furoindolizidinone 111, a direct hydrogenation of the ketone and the furan resulted in a mixture of the four different diastereomers (113-116), albeit the d.r. of the reaction was highly dependent on the employed catalyst. While Pd/C, Ru/C and RANEY®-Ni produced poor d.r., Rh/Al_2_O_3_ gave 113 as almost the sole product (d.r. = 96 : 0:1 : 3). The major contributor to the diastereoselectivity is the steric hindrance of the pyrrolidone ring junction which guides the hydrogen to the *exo* face of the molecule. However, if the ketone was reduced with NaBH_4_ to the ancillary alcohol 112 prior to the hydrogenation of the furan, the diastereoselective outcome was predominantly determined by haptophilic effects. The alcohol and the furan oxygen coordinate to the catalyst guiding it and consequently the hydrogen to the same face of the ring system. Therefore, 115 is formed as major diastereomer with an excellent d.r. = 93 : 7 and RANEY®-Ni as the most selective catalyst (Scheme 16D).

**Scheme 17:**
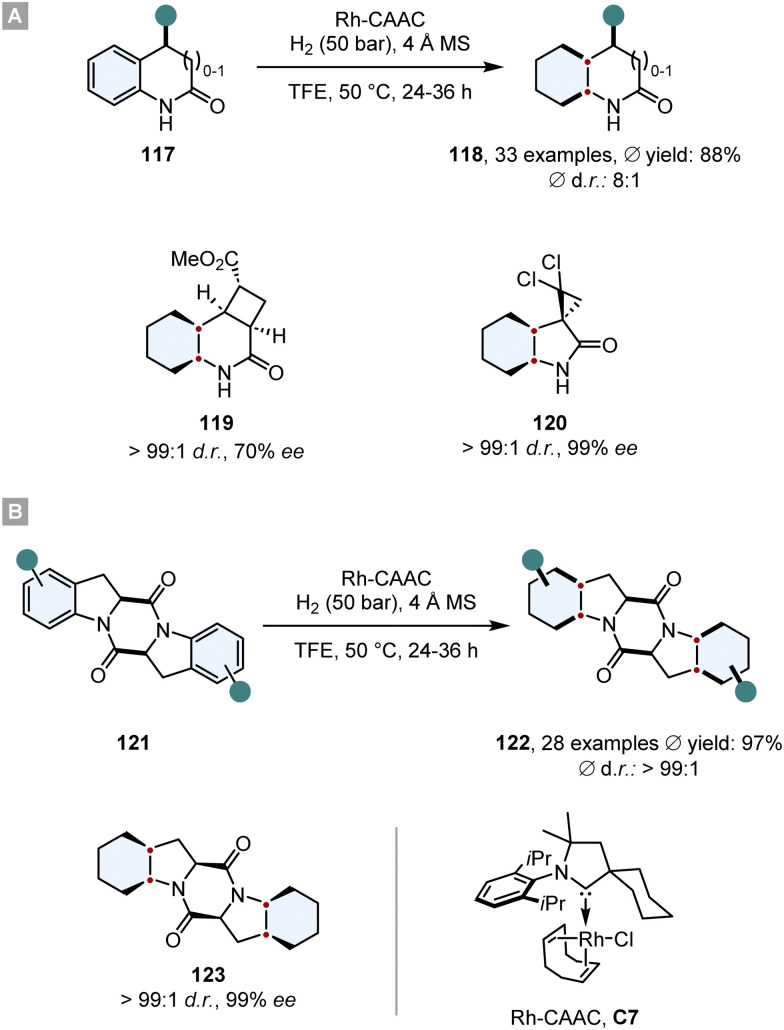
Asymmetric diastereoselective hydrogenations of (A) 2-oxindoles, 3,4-dihydroquinolones and (B) aromatic 2,5-diketopiperazines with a highly selective Rh-CAAC catalyst.^124,125^

Bach and co-workers disclosed highly diastereoselective hydrogenations of 2-oxindoles, 3,4-dihydroquinolones (117) and aromatic 2,5-diketopiperazines 121 in 2020 and 2022 (Scheme 17).^124,125^ As a catalyst was employed a rhodium complex, bearing a cyclic (amino)(alkyl)carbene (Rh-CAAC, C7). This complex decomposes under hydrogenation conditions into nanoparticles which are active for arene hydrogenation, but tolerate a plethora of reductively labile motifs such as ketones,^126^ fluorine,^127–129^ boron^130^ or silicon.^131^ Additionally, it displays a high diastereoselectivity towards the all-*cis* configurated product. Also, Bach and co-workers observed great functional group compatibility (fluorine, boron, silicon, amides, alcohols, amines, ethers, esters), good yields (*ϕ* 72%) and high levels of diastereoselectivity for the all-*cis* product 118 (for 2-oxindoles and 3,4-dihydroquinolones *ϕ* d.r. 89 : 11, for 2,5-diketopiperazines *ϕ* d.r. > 99 : 1). The great facial selectivity can be attributed to the steric repulsive effects of the substituents, shielding one side efficiently. Furthermore, this method was used in the synthesis of enantiomerically pure compounds 119 and 120. Especially, optically pure 2,5-diketopiperazines like 123 are a reoccurring scaffold in various natural and biologically active products.^132^ This underlines the utility of this method for its application in total synthesis of biologically relevant compounds.

**Scheme 18:**
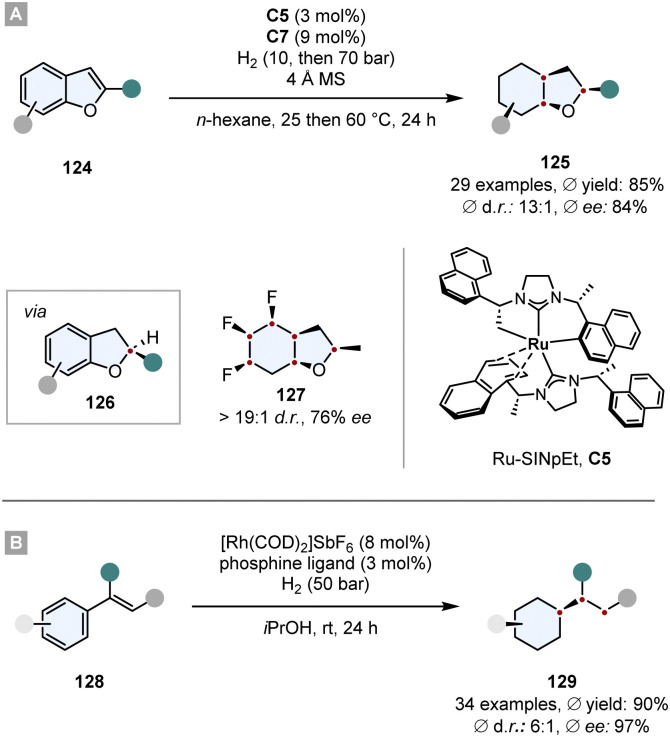
Hydrogenations employing relay catalysis to first set a stereocentre and then perform a diastereoselective hydrogenation.^133,134^

The same Rh-complex was used by Glorius and co-workers in a novel relay catalysis approach to construct enantiomerically pure octahydrobenzofurans 125.^133^ In a one-pot reaction, first the furan ring was reduced by chiral Ru-SINpEt C5 to set the stereocentre. For this step it is crucial that it proceeds at low temperature to (a) achieve a high ee and (b) prevent a racemic background reaction. At 25 °C the Rh-CAAC complex C7 does not form nanoparticles, which would hydrogenate the benzofuran completely to give a racemic mixture. After 3 h the temperature was elevated to 60 °C and the pressure to 70 bar to induce the formation of nanoparticles and the diastereoselective hydrogenation of the remaining benzene ring. The facial discrimination of the second step was obtained by the pre-set stereocentre. With this relay catalysis the authors showed a broad substrate scope, in general high yields (*ϕ* 85%) and diastereoselectivities (*ϕ* 93 : 7 d.r.), thus combining the great chemo- and diastereoselectivity of the Rh-CAAC complex C7 with the high enantioselectivity of the Ru-SINpEt C5. Additionally, it was possible to set up to six stereocentres in a single synthetic operation (Scheme 18A).

In a similar manner, Andersson and co-workers used relay catalysis for the asymmetric full saturation of vinyl arenes 128.^134^ They discovered that [Rh(COD)_2_]SbF_6_ as Rh-precursor can aggregate into nanoparticles potent enough to reduce benzene rings. Simultaneously, it can also form an irreversible complex with bisphosphine ligands that are known to hydrogenate enamides with a high degree of reactivity and enantioselectivity. Kinetic studies confirmed that the Rh-bisphosphine-complex fully hydrogenates the enamide in under 20 min and after an induction period of ∼1 h Rh-nanoparticles are formed that then hydrogenate the benzene ring. Essential for the success is a metal to ligand ratio of ∼2 : 1. In general, good yields, ees and d.r.s were obtained whereas the *in situ* set stereocentre efficiently shields one face of the benzene ring. Further utilisation of the method has been shown in the synthesis of a saturated analogue of Rasagiline, an anti-Parkinson's therapeutic (Scheme 18B).

## 5. Future and outlook

**Fig. 10:**
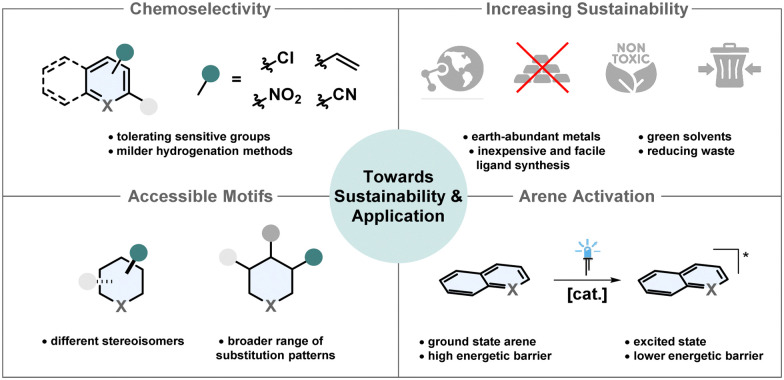
Illustration of future objectives to improve the sustainability and broaden the applicability of asymmetric arene hydrogenation.

In the past two decades asymmetric arene hydrogenation quickly evolved to a useful synthetic strategy to efficiently access highly valuable complex molecules. Despite the remarkable progress in the field, there are still remaining challenges to broaden the applicability in synthesis and with regard to more sustainable chemistry (Fig. 10).

### (1) Chemoselectivity

Especially the development of milder and therefore more chemoselective and more practical diastereoselective arene hydrogenation methods in recent years expanded the scope of valuable three-dimensional products significantly.^21,135,136^ Tolerance of reductively labile groups is of high importance, since these are either synthetic handles for further manipulations or important structural features of the product. For most arene hydrogenations various important but under reductive conditions sensitive groups like alkenes, alkynes, halogens, nitro- and nitrile-groups cannot yet be tolerated. Although more sophisticated methods to tackle this problem are developed,^56^ often the functional groups are in remote positions. There is still a demand to particularly tolerate these groups if they are directly attached to the arene that is being reduced.

### (2) Larger variety of accessible motifs

Depending on the (hetero)arene, the available methods for the asymmetric hydrogenation are limited to only a few substitution patterns. In particular, the enantioselective hydrogenation of (hetero)arenes mostly requires a substituent in a certain position and switching to different substitution patterns is not possible without a dramatic loss in enantioselectivity.^21^ Pyridines are highly available substrates and chiral piperidines are ubiquitous motifs in drug molecules.^137^ So far, direct access of chiral piperidines from pyridines requires a 2-substituent and only few patterns work sufficiently. Chiral auxiliaries in combination with heterogeneous catalysts are therefore needed for more diverse substitution patterns. Asymmetric hydrogenation of carbocyclic rings remains underrepresented. There is no efficient procedure for the enantioselective hydrogenation of benzene and also for naphthalenes only few methods are available.^13,21^ Furthermore, most asymmetric arene hydrogenations only enable access to one diastereomer, which is mostly the all-*cis* configurated one. Synthetic methods to access different diastereomers are desired.^21^ As demonstrated by Stoltz and co-workers, switching the diastereoselectivity even with the same catalyst only by adjusting the additive and the solvent is not elusive anymore.^63^ If one catalyst can be used to access different diastereomers or even enantiomers as shown by Dong, Zhang and co-workers^52^ it can lead to more sustainable chemistry since in some cases one enantiomer of the catalyst is easier to access.

### (3) Increasing sustainability

Solvents are the major factor of waste production in most chemical reactions.^138^ Consequently, it would be beneficial to either use solventless systems or solvents from renewable feedstocks. Since many hydrogenations rely on toxic and/or halogenated solvents, there is a potential to increase sustainability by substitution with greener alternatives.^16^

Additionally, highly active catalysts with a high TON and TOF are desired because these are usually expensive and laborious to recycle.^139,140^ Sophisticated ligand synthesis increases step-count, waste production and cost of the overall catalyst. Simple ligands that can be easily synthesised from the chiral pool are advantageous.^141^ Most asymmetric arene hydrogenations require catalysts based on rare and expensive metals like iridium, rhodium and palladium.^13^ Much more desirable and environmentally benign are catalytic systems based on cheap and highly available earth-abundant metals including 3d-metals.^142^ However, often these complexes are sensitive to air and not reactive enough to promote efficient arene hydrogenation.^143^ The investigation of new ligand designs, especially pincer ligands for 3d-metals, is crucial.^144^ Only very recently, Lan, Liu and co-workers reported their pioneering work of the first manganese catalysed enantioselective arene hydrogenation.^56^

### (4) Innovative arene activation

Efficient hydrogenation often requires activation of the substrate or the catalyst.^145^ Common strategies include the use of strong Brønsted acids, Lewis acids, halide-based reagents and other additives resulting in more by-products, lower atom economy and lower tolerance for functional groups.^145–147^ A new activation strategy which prevents the production of additional waste is an important goal to achieve more sustainable transformations. A yet not employed strategy in asymmetric hydrogenation is the activation with visible light. Chirik and co-workers recently achieved the visible-light mediated hydrogenation of anthracenes with an iridium catalyst at only 4 bar of hydrogen pressure. While the direct HAT from the metal-hydride to the anthracene is endothermic and unlikely to happen, the HAT from the excited state is exothermic and promoted efficient hydrogenation.^148^ Another example for the photoinduced (partial) hydrogenation of arenes was recently reported by Zheng, Wang and co-workers who used boron carbonitride as a metal free heterogenous catalyst to promote energy- and electron transfer to formally hydrogenate a vast scope of different hetero- and carbocyclic arenes.^149^ Future research in the field of photo-mediated arene hydrogenation has the potential to achieve milder and more sustainable asymmetric methods.

## Conflicts of interest

There are no conflicts to declare.

## Supplementary Material
